# Migration, refugees, and health risks.

**DOI:** 10.3201/eid0707.017733

**Published:** 2001

**Authors:** M Carballo, A Nerukar

**Affiliations:** Centre for Migration and Health, Vernier, Switzerland. mcarballo@icmh.ch

## Abstract

Migration both voluntary and forced is increasing all over the world. People are moving in larger numbers faster and further than at any other time in history. This is happening at a time when many countries are ill-prepared to deal with a changing demography and when policies and attitudes to population movement and immigration are hardening. The health implications of this are many, and, in some cases, illness and death rates associated with migration are exacerbated by a lack of policies needed to make migration a healthy and socially productive process. From a public health point of view, this is having and will continue to have serious ramifications for the people that move, the family they leave behind, and the communities that host the newcomers.


### Migration, Refugees, and Health Risks

**Manuel Carballo* and Aditi Nerukar†**
*International Centre for Migration and Health, Vernier, Switzerland; †Research International Centre for Migration and Health, Vernier, Switzerland

Migration—both voluntary and forced—is increasing all over the world. People are moving in larger numbers faster and further than at any other time in history. This is happening at a time when many countries are ill-prepared to deal with a changing demography and when policies and attitudes to population movement and immigration are hardening. The health implications of this are many, and, in some cases, illness and death rates associated with migration are exacerbated by a lack of policies needed to make migration a healthy and socially productive process. From a public health point of view, this is having—and will continue to have—serious ramifications for the people that move, the family they leave behind, and the communities that host the newcomers.

On Sunday June 18, 2000, 54 Chinese would-be immigrants suffocated to death while trying to enter the United Kingdom in a sealed truck designed to carry fruit from continental Europe. The path they took started in Fujian, a Province in southeastern China. From there they traveled to Beijing and then to Kazakhstan or Russia, the Czech Republic, Austria, France, and across the English Channel to Dover where they died.

They covered thousands of miles, and the trip cost each of them an estimated $30,000 as well as their lives. About 100,000 people from Fujian Province attempt to emigrate each year, most of them unofficially (illegally). Fujian is just one of thousands of areas throughout the developing world sending people who believe that a better quality of life and greater opportunities exist elsewhere. Modern methods of communication have made the world a far smaller place and introduced people everywhere to different concepts and increasingly shared values and expectations.

Migration is not a new phenomenon, of course. Early hunting and gathering societies migrated constantly, and nomadic herdsmen in many parts of the world still move routinely. The United States, Canada, and Australia were built on migration, and most European countries were saved by being able to send millions of people to other places when confronted with massive agricultural, political, or economic crises.

Today as many as 190 million people are thought to cross borders every year, and migration has become an integral and inevitable part of global social and economic development. More importantly, the possibility of moving elsewhere is becoming an increasingly key part of how people view the world they live in. Growing poverty in some regions is pushing more and more people to look for opportunities elsewhere at the same time as politicians and social forces are trying to prevent more immigration. The public health implications of this emerging dynamic, so replete with political contradictions, are enormous.

At the same time that voluntary migration is increasing, so are far more tragic types of uprooting and displacement. Wars, natural disasters, and complex emergencies that destroy social and cultural infrastructures are affecting more people than ever before, because as the world's population grows and becomes more concentrated, so does the number of people at risk for being affected by these events.

Despite the time and effort spent on conflict prevention and resolution, over the last 15 years alone over 36 countries have been involved in conflicts of one kind or another. These conflicts have collectively been responsible for uprooting over 60 million people—more than the combined populations of at least 13 Western European countries. Such are the vagaries of peace accords and resettlement policies that many of these refugees will remain stateless and homeless for years and, in some cases, generations to come.

Many of the same factors that influence those who migrate for economic reasons also influence the movement of refugees. Better communication and easier and faster transport systems have increased the number of places they can settle. Thus, within months of being forced from their homes in Bosnia, refugees were taken in as far afield as Malaysia, Australia, Canada, and the United States as well as many other countries closer to home. The same situation occurred with Kosovar Albanians some 7 years later, and by the end of 1999, these refugees were dispersed over at least 60 countries.

Were these mass movements simply demographic phenomena the problem might be of purely academic interest. However, the reality is that, with respect to both voluntary and forced migration, deaths occur. The 54 Chinese people who died in the back of the truck in Dover, England, were a small tip of a large iceberg. Mexicans trying to enter the United States take the same risks every day and their mortality rate is probably just as high. From Valencia, Spain, down to the Straits of Gibraltar police patrols regularly find the bodies of Africans who drowned trying to enter Europe through its southernmost door. Albanians trying to get to Italy risk meeting the same fate in the Adriatic Sea.

The number of migration-related deaths will continue to grow if policies designed to keep people out of countries are more stringently enforced just at the time when pressure on them to go to new countries is increasing. However, in addition to these mortality statistics and human rights

Address for correspondence: Manuel Carballo, International Centre for Migration and Health, 11 Rte Nant d'Avril, 1214 Vernier, Switzerland; fax: (41 22) 783 1087; e-mail: mcarballo@icmh.ch questions, other public health considerations need to be addressed. Because migrants are typically poor people moving from poor economic environments, they carry with them the health profiles that result from poverty. Their understanding of health comes from having to adapt to poor ecological conditions along with limited possibilities for change and control over their own life.

Despite the magnitude of the migration process now underway, many European countries have been relatively unprepared to deal with it, and few have formulated policies needed to make immigration a healthy and socially productive process. Health indicators suggest that migrants in Europe are at considerably higher risk for contracting a number of diseases than nonmigrant populations in the same countries.

### The Process of Migration

Migration, even under the best of conditions, involves a series of events that can be highly traumatizing and that can place migrants at risk. The process involves uprooting, being separated from family and traditional values, and being placed in new social and cultural situations where job and legal security may be minimal. For many migrants, social integration is rarely easy and for some impossible.

Resistance to their presence—even when their work skills are needed—often places immigrants on the periphery of society. Resistance to their participation in society results from language problems and culturally-defined behavior that often reinforce stereotypes and prejudices. Not only are migrants themselves affected, but, in many situations, their children are also discriminated against. Studies of this phenomenon, carried out by the International Centre for Migration and Health in the European Union (EU), indicate that children of migrants may be at high risk for drug abuse because they use drugs to demonstrate their rejection of, and exclusion from, so-called mainstream society.

The labile nature of health and health-related behavior makes people highly susceptible to the social dynamics and physical environments they live in, especially when there is little or no social support to compensate for exclusionary attitudes and poor working and living conditions.

### The Demographics of Infectious Diseases in Migrant Workers

The incidence of new cases of tuberculosis infection (TB) in 9 EU countries fell from 34.8 out of every 100,000 persons in 1974 to 14.3 out of every 100,000 in 1995 in all 15 EU member states. In Denmark, the incidence of new cases increased steadily over the past 5 years, and the proportion of cases in foreign-born persons rose from 18% in 1986 to 60% in 1996. In England and Wales, approximately 40% of all TB infections are estimated to occur in people from the Indian subcontinent. In the Netherlands, where the incidence of TB rose 45% between 1987 and 1995, over 50% of known cases of infection occurred among immigrants. The TB profile in Germany and France is similar, and migrants are 3 times and 6 times, respectively, more likely to be diagnosed with the disease than are nonmigrants.

The irony of these figures is that, in many countries, the working and living conditions of migrants may be creating risk factors for them to contract TB. Social exclusion and language barriers, as well as cultural attitudes to seeking healthcare, often render the biomedical risks even greater. Migrants may may not be eligible for national health insurance plans, or they simply may not know where and how to seek help. In some cases, when immigrants come from high TB-endemic areas, they may have high tolerance levels for discomfort and not seek help as readily as the native population of EU countries.

Much the same can be said of hepatitis A, which, like TB, is a disease of poverty and endemic in most developing countries. Exposure to it as a result of living conditions in some EU countries cannot be excluded from the risk situations that migrants face.

The link between hepatitis B and HIV/AIDS when unprotected sexual contact is a factor presents many psychosocial questions, namely those surrounding migration and social integration. To understand this better, however, it is perhaps worthwhile to take into account the risks that travelers and tourists take because, in many ways, their behavior is often dictated by similar conditions as those experienced by migrants.

In one region of the United Kingdom, people traveling abroad accounted for more than 6% of all reported cases of hepatitis B in 1981, and from 1990 to 1994 when travel had become more popular, the proportion had more than doubled. In Germany, where an estimated 50,000 new cases occur per year, at least 7,500 are estimated to be associated with travel by Germans to other countries.

Many of these same underlying factors contribute to migrants' risk of contracting HIV/AIDS. Many EU countries require migrant workers to travel alone and leave spouses and partners behind. This, almost inevitably, places migrants at risk for unsafe sexual behavior, contact with sex workers, and infection with sexually transmitted diseases (STDs).

Yet the risk of migrant workers contracting STDs other than HIV/AIDS is an issue that has not been addressed by most national health authorities. In Belgium, illness from STDs is reported to be significantly higher among unmarried male immigrants than it is among Belgian men in general. Similarly, in Sweden, where there has been an overall decrease in the incidence of new cases of STDs, the number of cases among foreign-born persons is superseding the number among men born in Sweden.

The incidence of HIV/AIDS is also higher among migrants living in Sweden, especially those from Africa, than among Swedish nationals. In Germany a similar pattern is emerging, and it is becoming clear that the risk of HIV/AIDS among migrants reflects the global epidemiologic pattern of HIV/AIDS. Thus, in Germany where the number of AIDS cases among migrants has risen in recent years and represents approximately 14% of all reported cases, it is most commonly found among migrants from Africa, North America, Asia, and Latin America, but migrants from Turkey and Eastern Europe have been minimally affected. The picture is a complex one, however, and in Italy the risk of HIV/AIDS in migrant populations appears to be lower than among nationals.

### Reproductive Health Among Migrant Women

Communicable diseases are by no means the only health problems to which migrants appear to be highly susceptible. Reproductive health in general, especially among women, seems to be affected by changes in social and economic environment, access to health care, changes in sexual behavior, and social status.

Difficult pregnancies and pregnancy-related illness among migrants are problems throughout the EU. In the United Kingdom, babies of Asian mothers tend to have lower birthweights than other babies, and perinatal and postnatal mortality rates are higher among immigrants born in Pakistan and the Caribbean than in the general population.

Data from Belgium indicate that in 1983 the highest perinatal and infant mortality rates were for babies born of immigrant women from Morocco and Turkey. By 1993, the situation had improved substantially for the domestic Belgian population and for Moroccan immigrants, but among Turkish immigrants high perinatal and infant mortality rates persisted and in 1993 were still 3.5 times higher than those for Belgians.

In Germany, perinatal and neonatal mortality rates are consistently higher in foreign-born groups, especially Turkish immigrants, than in the population as a whole. The rate of perinatal mortality for babies born to German mothers is approximately 5.2% and among nonnationals approximately 7%, and the incidence of congenital abnormalities and maternal mortality is also higher among immigrants.

In Spain, premature births, low birthweight, and complications of delivery are especially common with infants born of women who have immigrated from sub-Saharan Africa and Central and South America. African immigrant women giving birth in hospitals, for example, have an incidence of premature births almost twice as high as in Spanish women, and low-birth-weight rates are also approximately double those of women born in Spain.

Over 8% of babies born to women from Central and South America are underweight and 6.3% are born prematurely. Unwanted pregnancy and poor knowledge about contraception and where to get contraceptive devices and advice on contraception are common problems among immigrant women, and requests for abortions tend to be twice as common among them as Spanish women, especially those coming from North Africa and the sub-Saharan region.

### Occupational Health and Safety Among Immigrants

Migrants tend to take jobs that are temporary, require few skills, and are largely unattractive to local labor forces. Many jobs that are available, such as those in mining, construction, heavy manufacturing, industry, and agriculture, often involve poor environmental conditions and lack of safety.

Because temporary labor markets are often seen as too short-term to justify major investments in training, employers tend not to require instruction and careful supervision. Language obstacles, poor communication, lack of familiarity with some of the technology used, and different attitudes to work safety all contribute to work-related risks.

The number of industrial accidents and injuries is higher among migrant workers than among citizens in France and Germany, especially those who work in construction and public works types of jobs. Over 30% of all accidents resulting in permanent disabilities involve non-nationals. Data from Belgium similarly indicate that Moroccan and Turkish workers in heavy industry have a higher incidence of accidents than Belgian nationals and suffer more secondary psychological sequelae.

In the agricultural sector, unprotected exposure to pesticides and other chemical products is a common problem, and chronic exposure to them has been linked to depression, neurologic disorders, and miscarriages among migrant workers in Spain. The incidence of other injuries among people working in greenhouses is also high, and muscular disease, dehydration, and heart complaints linked to high temperatures are common. Few agricultural workers receive much safety training, and few use effective protection. Because many agricultural businesses are small and harvests seasonal, health authorities tend to have limited access to them, and little routine evaluation of working conditions and safety takes place.

### Domestic Accidents and Poisonings Among Immigrant Children

Immigrants also tend to be more vulnerable to other types of accidents. In Germany, non-German children 5 to 9 years old have more traffic and other accidents than German children. Children of Moroccan and Turkish migrant workers in the Netherlands also have more domestic accidents such as poisonings and burns, as well as traffic accidents, than Dutch children. Poor quality housing is often a risk factor, and in France, lead poisoning from paint in old, poorly maintained houses is a major problem for migrant children. The frequent absence of one or both parents due to heavy work schedules, along with poor childcare alternatives available to immigrants also contributes to putting children of migrants at risk.

### Psychosocial Issues Among Immigrants

Underlying many of these health issues are the psychosocial determinants of health-related behavior. Whether migration is planned or not, voluntary or forced, some degree of stress is always involved. Migration means breaking with family, friends, and established social networks, departing from traditional routines, value systems, and accepted ways of behaving and having to adapt to new social and psychosocial environments.

Despite the potential magnitude of the problem, the psychosocial health of migrants remains poorly addressed. Relatively little is known about the dynamics involved or about what should and can be done to prevent or manage mental health problems related to migration. In the EU, where both the number of immigrants and the amount of internal movement is increasing, social integration and acculturation (defined as the gradual adoption of the identity, values, behaviors, and attitudes of the host community) are linked to a number of mental health problems.

Language also plays an important role in mental health, and barriers to good communication compound feelings of isolation and being "unwanted." The capacity to communicate can influence healthcare-seeking behavior, underreporting, poor explanation of health problems and symptoms, inappropriate diagnoses and the capacity of immigrants to comply with treatment regimens.

Some EU countries have adopted settlement policies that stress the social dispersal of ethnic minorities and immigrants in order to integrate them more quickly into "mainstream" society. There is little evidence that this has been effective; indeed, the feelings of isolation that follow social dispersal are often detrimental to both mental health and social integration. This has been the case with Vietnamese refugees in Finland, where younger immigrants were more able than their parents to adopt Western values and behaviors, but their "success" prompted anxiety and depression among mothers who felt they were "losing" their children.

Among rural Turkish workers in Amsterdam, only a few are able to speak Dutch, and the capacity to function and integrate into mainstream society has often been limited. Mental health problems such as neuroses are common, and over half of these immigrants say they worry and often regret their decision to move away from home. Less than 50% of those who are married are able to bring their families because work contracts and conditions do not allow it.

In Germany, an estimated 13% of immigrants seen for depressive disorders develop problems during their initial 12 months away from home. Another 25% tend to have problems within the following 2 to 5 years. Many immigrants say they "long for home" and report exaggerated memories of familial events, the ways they lived, and things they experienced as children. Fantasies about home and returning home are often described as "migrant's opium," and although these responses are not necessarily serious, they are often psychologically debilitating. In no case is this more true than when families are forced to migrate and are violently dispersed. High levels of anxiety among Bosnian refugees in Austria have been linked to their inability to trace lost family members, and this is felt to have become one of the main barriers to any real improvement in their overall quality of life in many host countries.

The relatively high incidence of depression among immigrants and their children in many EU countries has also been associated with high rates of suicide, possibly linked to unemployment. In the Netherlands, where the unemployment rate among migrants in 1994 was 31% compared to 13% for Dutch nationals, the suicide rate among children of immigrants was also considerably higher than in the general population. In Rotterdam, children of Turkish immigrants were five times as likely as Dutch children to commit suicide and Moroccan children three times as likely. Children, particularly girls, of Surinamese immigrants had a suicide rate 27.6 times higher than that of Dutch children. In the United Kingdom, suicide rates for women from the Indian subcontinent are also markedly higher than for men and are highest among girls ages 15 to 24. On the whole, suicide among this immigrant group is twice as high as the national average and in the 25 to 34 year old age group, 60% higher. Attempted suicide among young women from south Asia is also high.

For many people, migration and resettlement result in social isolation and loneliness. This is especially so when people move alone. The relief found in temporary friendships and supportive social environments at ethnic bars often heightens dependency on alcohol and other substances, and alcohol abuse is a frequent problem among single immigrants.

### Substance Abuse Among Immigrants

Alcohol abuse among male Indian immigrants, especially Sikhs, is increasing and reflected in higher mortality rates associated with cirrhosis of the liver, which are twice as high as they are for men born in England. This trend is not typical of all Asian immigrants, however; Pakistani males (who tend to be Moslem) are far less likely to consume any alcohol. Irrespective of religious background, women from the Indian subcontinent are also unlikely to consume alcohol, and their death rates from alcohol-related causes are similar to those for English women.

Drug abuse also may be an emerging problem among immigrants. In the United Kingdom, immigrant drug users who have tested HIV-positive have begun to attract attention, and the migration of drug users coming to the UK in search of better living conditions has become a serious problem.

In Amsterdam, about half of people using methadone bus outreach programs are non-Dutch, and about a quarter of all young women who leave drug Youth Advice Centers prematurely are from other countries; 45% of detainees in youth penitentiaries are migrant children. The reasons for drug abuse among children of immigrants vary considerably, but in France, it is seen as a manifestation of social marginalization and an expression of anger at the problems of integration. A study of psychological stress and coping among Greek immigrant adolescents in Sweden tends to confirm this, and similar observations have been reported in Germany.

Patterns of drug abuse among migrants and refugees, however, may not necessarily be different from those of local populations, and evidence is growing that what really differentiates immigrants and their children from nationals, is the limited ability or unwillingness of immigrants to use local health and social services for fear of being reported or because they do not receive culturally sensitive support.

### Conclusion

Migration nurtures economic development, encourages interdependence between countries and regions, and also provides important links for the exchange of resources between the EU and other countries. The economic need for this movement of human resources is well-established, and it is unlikely that the trend will diminish of its own accord in the foreseeable future. Indeed, the free movement of people that has become a basic principle of the EU will no doubt be extended to include other countries as they are admitted to the Union.

Despite the magnitude of the public health issues involved, relatively little attention has been given to the role of migration in changing the epidemiologic profile of receiving communities or the impact migration and resettlement have on the health of immigrants. Even less attention has been paid to conditions possibly linked to poor health in the context of migration. National health statistics rarely reflect the process or its implications, and there has been relatively little interest in the phenomenon by health and social scientists. What data are available tend to be from small studies and anecdotal reports. They nevertheless indicate that the health circumstances that characterize uprooting and migration merit more consideration.

Much, of course, depends on the type of population movement, why and where it occurs, and the profiles of the people involved. However, the fact that most migrants move because of the "push" of poverty rather than the "pull" of better living conditions means that many of them inevitably come from socioeconomically deprived backgrounds where they are not able to provide a good quality of life or health for themselves. Migrants moving because of poverty arrive with health profiles typical of those in their previous surroundings. Poverty breeds diseases of poverty no matter where or when it exists.

The range of health issues that can be associated with migration is inevitably broad. It includes communicable and noncommunicable diseases, injuries associated with work environments, and psychological problems. All or any of them can be debilitating to the health of migrants and their families. They can also have serious consequences for societies and communities into which they move and work.

Communicable and noncommunicable diseases, as well as occupational injuries, psychosocial problems, and family breakdown, can all be equally important with regard to the care and treatment required to deal with them and the work and school days that can be lost because of them. The health of migrants thus has social and economic consequences for host countries as well as for migrants and their families.

The process of migration within and into the EU includes changing and emerging trends in health. TB is a disease of poverty, and it is not surprising that migrants from poor countries are at high risk of contracting it. Much could be done to avoid TB by ensuring better standards of living for low income migrants who must often deal with poor housing and overcrowded conditions. Early identification and treatment of TB is also important and screening should be seen as a way of identifying people who need treatment. However, associating health screening with the possibility of expulsion does little to encourage migrants to participate and indeed may defeat the whole purpose of it. Illegal immigrants are possibly at particularly high risk of TB and reluctant to be tested for fear of being expelled. Screening for other diseases such as hepatitis, which also constitutes a growing threat, could also be useful if it is done in a constructive way.

Problems associated with maternal and child health among migrants have long been a matter of concern in most EU countries. Migrant women make poor use of contraceptive services and have more difficult pregnancies than other women. They have more low-birth-weight babies and tend to deliver prematurely more often. In some countries, migrant women also terminate pregnancies more often than other women. In some migrant communities, women are still regarded as second-class citizens. Different cultural practices within families and attitudes toward women often seriously limit their access to, and use of, antenatal care and other services. Conflicting pressures are brought to bear on women caught between traditional domestic values and practices and those of the social environment they work and live in and thus confront women with difficult psychological barriers.

Psychological stress may also contribute to the problems immigrants face, and the deprivation, employment difficulties, and problems of cultural and social adaptation experienced by migrants deserve more consideration. Early identification of people at risk and counseling for these people are urgently called for.

The fact that migrants are often qualified for only low-skilled jobs also means they are often confined to high-risk and irregular occupational settings. The fact that they come into them with little previous experience and receive little training and safety support means that they are often exposed to health problems and accidents associated with low-skilled jobs.

For ethical as well as public health reasons, occupational hazards for immigrants is an area that calls for much more attention than it has received to date. The fact that documentation in this area is so weak is, in itself, indicative of the little attention it has been given. As a first step, better training given by people who speak the languages of migrants would be an important contribution to solving these problems.

Culture conflict is a common and serious problem in migration. It affects people in different ways, some more overtly than others. To what extent culture conflict or clash is linked to mental health problems reported among migrants is not clear, but there is reason to believe that it is a factor. Just as with other health indicators, however, there is a paucity of information on the incidence and prevalence of mental health problems among migrants. Available data suggest that cultural background plays an important role in predisposing some immigrants to some diseases such as depression, chronic anxiety, and neuroses. Alcohol and drug abuse may also be used as coping responses that expose migrants to other health problems such as HIV/AIDS. However, in general, the trauma and exclusion that all immigrants face increase their risk of behaviors that, in turn, increases their susceptibility to all diseases.

